# Contribution of Retzius-sparing robot-assisted radical prostatectomy to the mechanism of urinary continence as demonstrated by dynamic MRI

**DOI:** 10.1038/s41598-023-30132-x

**Published:** 2023-02-18

**Authors:** Yoshifumi Kadono, Takahiro Nohara, Shohei Kawaguchi, Renato Naito, Suguru Kadomoto, Hiroaki Iwamoto, Hiroshi Yaegashi, Kazuyoshi Shigehara, Kouji Izumi, Kotaro Yoshida, Toshifumi Gabata, Atsushi Mizokami

**Affiliations:** 1 Department of Integrative Cancer Therapy and Urology, Kanazawa University Graduate School of Medical Science, 13-1 Takara-machi, Kanazawa, Ishikawa 920-8640 Japan; 2 Department of Radiology, Kanazawa University Graduate School of Medical Sciences, Kanazawa, Japan

**Keywords:** Oncology, Urology

## Abstract

Retzius-sparing robot-assisted radical prostatectomy (RARP) has been reported to exhibit better postoperative urinary continence, but the reasons behind this are unknown. This study included 254 cases who underwent RARP and underwent postoperative dynamic MRI. We measured the urine loss ratio (ULR) immediately after postoperative urethral catheter removal and investigated its affecting factors and the mechanisms. Nerve-sparing (NS) techniques was performed in 175 (69%) unilateral and 34 (13%) bilateral cases, whereas Retzius-sparing in 58 (23%) cases. The median ULR early after indwelling catheter removal in all patients was 4.0%. The multivariate analysis was performed on factors that reduce ULR and found that the following factors were associated with ULR: younger age, NS and Retzius-sparing, which were significant. Additionally, dynamic MRI findings showed that membranous urethral length and the anterior rectal wall movement toward the pubic bone during abdominal pressure were significant factors. The movement observed on the dynamic MRI during abdominal pressure was thought to reflect an effective urethral sphincter closure mechanism. Long membranous urethral length and an effective urethral sphincter closure mechanism during abdominal pressure were considered effective for favorable urinary continence after RARP. NS and Retzius-sparing were clearly shown to have an additive effect in preventing urinary incontinence.

## Introduction

Radical prostatectomy (RP) is the standard treatment for localized prostate cancer (PC), and robot-assisted RP (RARP) has been widely performed in recent years. Urinary incontinence is a complication after RP, which impairs the quality of life (QOL) and has is yet to be overcome^1^. The main cause of urinary incontinence after RP is thought to be stress urinary incontinence (SUI)^2^; however, the mechanism is not fully understood. Recently, many anatomical studies that use magnetic resonance imaging (MRI) have reported that membranous urethral length (MUL) affects postoperative urinary continence, which may be related to resting urethral closure pressure^3–7^. The mechanism of SUI has also been investigated using transperineal ultrasound and dynamic MRI, and several studies reported on the relationship between pelvic anatomy changes during abdominal pressure and postoperative urinary incontinence^8–11^. Additionally, many surgical techniques have been reported to reduce postoperative urinary incontinence. Among them, many have reported that the Retzius-sparing RARP (RS-RARP), which was reported by Galfano et al., is favorable for postoperative urinary continence^12–14^. Additionally, nerve-sparing (NS) techniques have been reported to work well for postoperative urinary continence^15–17^. Pelvic floor muscle training (PFMT) has been reported as effective in reducing urinary incontinence after RP to prevent SUI in women, and the results of several randomized controlled trials (RCTs) have been analyzed, including effective teaching methods for pelvic floor muscle exercises and combined biofeedback^18^.

Urine loss ratio (ULR) after catheter removal is a significant determinant of urinary continence after RP^19^. This parameter could have clinical usefulness to estimate future recovery of urinary continence^20^. The present study examined the factors that influence early postoperative ULR using postoperative dynamic MRI, along with the effect of changes in pelvic anatomy during abdominal pressure. Additionally, the impact of NS and RS-RARP on postoperative ULR and the relationship between each technique and dynamic MRI findings were examined.

## Methods

### Patient population

This study enrolled patients with clinically localized PC who underwent RARP performed by a single surgeon at Kanazawa University Hospital (Japan) from June 2016 to February 2022, for whom necessary pre- and postoperative data were collected. The study protocols were approved by the Medical Ethics Committee of Kanazawa University (approval no. 2016-022(2174)). All patients provided written informed consent, and all data were prospectively collected. All methods were performed following relevant guidelines and regulations.

### Surgical technique

Conventional RARP (C-RARP) was performed via a transperitoneal anterior approach. RS-RARP was introduced in July 2017, and the surgeon chose whether to perform C-RARP or RS-RARP for subsequent cases. RS-RARP was similarly performed to the technique described by Galfano et al.^12^. NS procedures were performed depending on cancer status. Urethral catheters were removed 6–8 days postoperatively after cystographic evaluation.

### Pelvic floor muscle training

A PFMT pamphlet was handed out at the outpatient clinic, and the outpatient staff gave a verbal explanation of the procedure. The patient was instructed to perform PFMT 5–10 times a day in the supine or seated position starting 1 month preoperatively, mainly by repeating the anal tightening exercise at quick intervals 5 times and continuing the exercise of tightening for approximately 5 s and then relaxing slowly 5 times in one set^21^. The staff provided re-education as appropriate if a patient complained of insufficient understanding.

### Dynamic MRI and study parameter measurements

**Figure 1:**
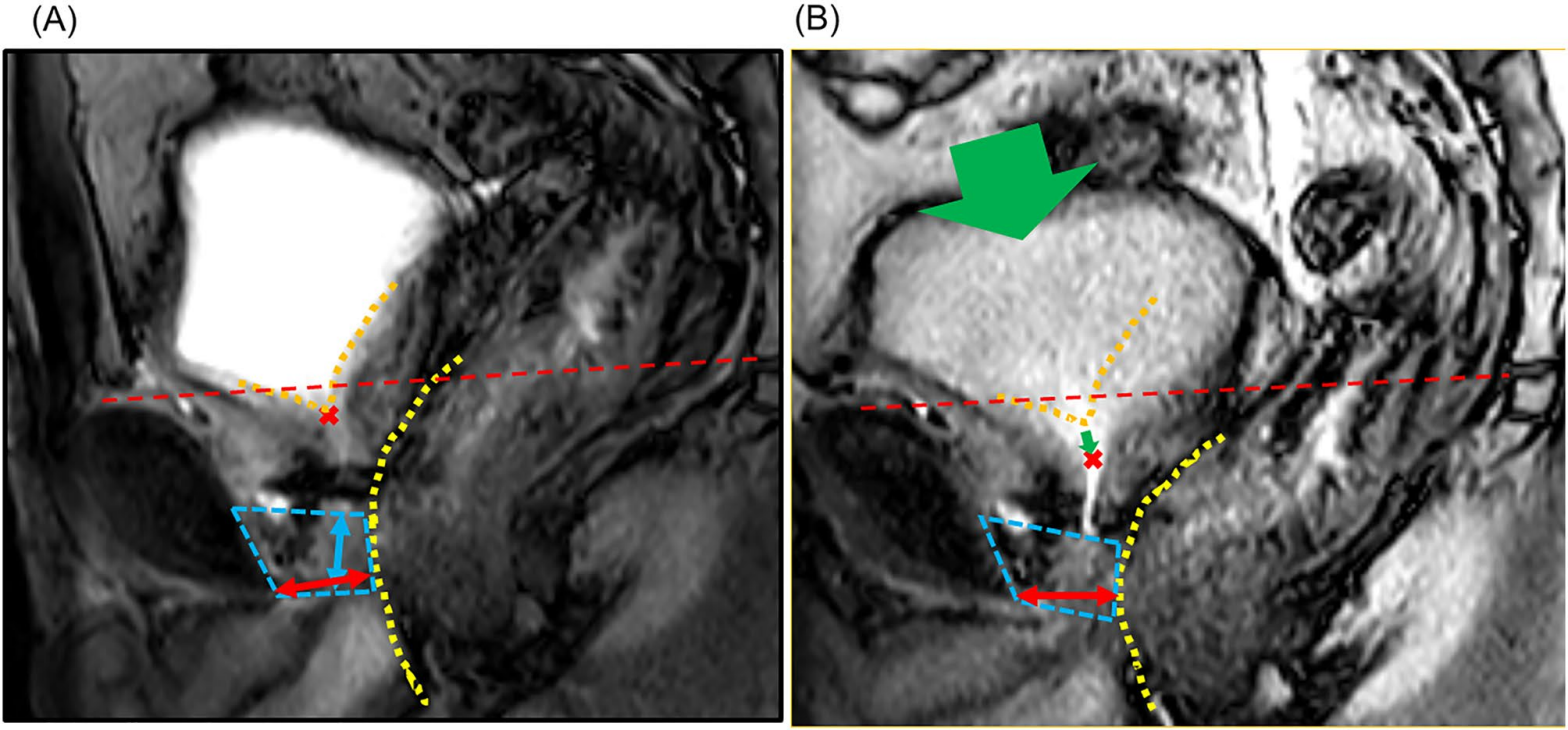
Dynamic mid-sagittal MRI after robot-assisted radical prostatectomy (RARP): at rest (**A**) and with abdominal pressure (**B**). The distance from the lowest point of the pubic bone to the anterior edge of the rectal wall (PB-AR, two-headed red arrow) was measured at rest and with abdominal pressure (large green arrow). The external urethral sphincter is indicated by the box surrounded by the blue dashed line. The anterior edge of the rectal wall is indicated by a yellow dashed line. The bladder base at rest is indicated by an orange dashed line. Each red cross is each urethrovesical junction (UVJ) at rest (**A**) and with abdominal pressure (**B**). The UVJ is positioned using the line that connects the superior border of the pubic bone and the lower sacrum (red dashed line) as an index. The small green arrow indicates UVJ movement.

MRI was performed within 1 week of postoperative indwelling catheter removal. Employed MRI machines for scanning were the 1.5-T or 3.0-T MR system (Signa Premier or Signa HDx; GE Medical Systems, Waukesha, WI, USA or Ingenia, Philips Healthcare, Best, The Netherlands) with a multichannel anterior array coil combined with a multichannel posterior table coil. The MRI was performed without urinating for 30 min to 1 h before the examination to allow the bladder to be filled up to approximately 100 ml. The patients were instructed to defecate before dynamic MRI. Multiplanar T2-weighted axial section imaging was performed to create an appropriate sagittal section for the prostatic urethra. Sagittal dynamic MRI was performed with one continuous image per second in the supine position for a total of 20 frames at rest and during the abdominal pressure phase using a fast spin-echo sequence with the following parameters: repetition time/echo time: 1500–3716 ms/85–104 ms; flip angle: 90°; slice thickness: 6 mm; field of view: 300 mm; and imaging matrix: 224–352 × 156–224. Figure 1A,B show the dynamic mid-sagittal MRI after RARP, which was performed at rest (Fig. 1A) and with abdominal pressure (Fig. 1B). Figure 1A illustrates the measurement of the external urethral sphincter thickness, defined as the shortest distance from the lowest point of the pubic bone to the anterior edge of the rectal wall (distance from the pubic bone to the anterior rectum: PB-AR) at rest, the mid-sagittal MUL, and the point of urethrovesical junction (UVJ). Figure 1B shows the point of greatest anterior rectal wall migration during abdominal pressure. Figure 1B shows similar PB-AR measurements at abdominal pressure. The PB-AR at rest was subtracted from the PB-AR at abdominal pressure to define the PB-AR change (mm), with shortening (the anterior wall of the rectum moved closer to the pubic bone) indicated as a plus, and elongation (the anterior wall of the rectum moved away from the pubic bone) is indicated as a minus. The UVJ was positioned using the line that connects the superior border of the pubic bone and the lower sacrum as an index (Fig. 1). UVJ movement (mm) was defined as the distance the UVJ moved from the resting measurement position toward the foot (i.e., the plus direction [positive]) and toward the head (i.e., the minus direction [negative]) during abdominal pressure. The micturition volume (MV) and weight of urine loss (UL) in the pads were separately assessed daily after catheter removal. The UL ratio (ULR) was calculated using the formula UL/(UL + MV). The ULR is calculated as the average ULR during 3-day hospitalization starting the day after the indwelling urethral catheter is removed. For cases discharged 3 days prior to indwelling catheter removal, the average is calculated over the 1–3 days prior to discharge. The ULR was divided into two parts by the median, with low ULR defined as below the median and high ULR defined as more than the median.

### Statistical analyses

Categorical variables for calculating incidences and percentages and continuous variables are presented as medians and interquartile ranges. The chi-square test was used for categorical variables, whereas the Mann–Whitney U test was for continuous variable comparisons. Factors that affect ULR were examined using logistic regression analysis. First, various univariate analyses were performed, followed by multivariate analyses of the influence of surgical technique and MRI findings, respectively. All data analyses were performed using Statistical Package for the Social Sciences for Windows (SPSS Inc., Chicago, IL, USA). *P*-values of < 0.05 were considered statistically significant.

## Results

### General characteristics

Of the 351 performed RARP cases during the study period, 254 cases were examined for which collecting the necessary data for pre- and postoperative analysis was possible. The studied cases were those after > 200 RARPs had been performed at our hospital, and RS-RARPs included the initial cases. Table 1 shows the background of the study cases. NS procedure was performed in 175 (69%) unilateral and 34 (13%) bilateral cases. Retzius-sparing was performed in 58 (23%) cases. The median ULR early after the indwelling catheter removal in all patients was 4.0%. Early postoperative dynamic MRI findings showed a median MUL of 11 mm, median UVJ movement due to applied abdominal pressure of 1 mm, and median PB-AR change of 1 mm.

**Table 1:** Clinicopathological characteristics of robot-assisted radical prostatectomy.

	Median (IQR) or n (%)
Number of patients	254
Age, years	67 (64–71)
Body mass index	23.6 (21.9–25.6)
Prostate specific antigen, ng/ml	6.6 (5.0–9.2)
Biopsy Gleason Grade Group	
1	54 (21%)
2	72 (29%)
3	57 (22%)
4	61 (24%)
5	10 (4%)
Clinical stage	
≦ T2	247 (97%)
T3 ≦	7 (3%)
D'Amico risk classification	
Low	48 (19%)
Intermediate	112 (44%)
High	94 (37%)
NADT	
No	235 (93%)
Yes	19 (7%)
IPSS total score	9 (6–14)
ICIQ-UI SF total score	0 (0–2)
Nerve-sparing	
Non	45 (18%)
Unilateral	175 (69%)
Bilateral	34 (13%)
Surgical time, min	253 (212–278)
Console time, min	177 (151–214)
PLND	
Non	184 (72%)
Limited	47 (19%)
Exteded	23 (9%)
Retzius-sparing, Yes	58 (23%)
Bleeding, mL	100 (30–150)
Blood transfusion, Yes	0 (0%)
Clavien-Dindo classification	
Grade 2 or less	254 (100%)
Grade 3 or grater	0 (0%)
Removed prostate volume, gr	39.5(31.0–49.0)
Catheter indwelling duration, days	7 (7–7)
Positive surgical margin	54 (21%)
Extraprostatic extension	39 (15%)
Urine loss ratio, %	4.0 (0.5–19.6)
Membranous urethral length, mm	11 (9–12)
UVJ movement, mm	1 (0–3)
PB-AR change, mm	1 (0–3)

*IQR* interquartile range, *ICIQ-UI SF* International Consultation on Incontinence Questionnaire-Urinary Incontinence Short Form, *IPSS* International prostate symptom score, *NADT* neoadjuvant androgen deprivation therapy, *PB-AR* distance from pubic bone to anterior rectum, *PLND* pelvic lymphnode dissection, *UVJ* urethrovesical junction.

### Investigation of factors that affect postoperative urinary incontinence

First, the correlations between factors that affect postoperative urinary incontinence were examined, with a significantly negative correlation between Retzius- sparing and UVJ movement (correlation coefficient [CC]: − 0.324, *p* < 0.001), and a significantly positive correlation between Retzius- sparing and PB-AR change (CC: 0.464, *p* < 0.001). A significantly negative correlation was found between UVJ movement and PB-AR change (CC: − 0.488, *p* < 0.001). Next, factors that affect the ULR in the early postoperative period were examined (Table 2). The median (interquartile range) for low and high ULR was 0.5% (0.4%–1.4%) and 19.6% (10.2%–40.2%), respectively. The logistic regression analysis was performed for each factor to examine its effect on ULR. The univariate analysis revealed that younger age, Retzius- sparing performance, longer MULs, more cephalad UVJ movement during abdominal pressure, and shorter PB-AR during abdominal pressure were statistically significant factors that reduce urinary incontinence (Table 2, Univariate). Next, we performed a multivariate analysis of whether NS or Retzius- sparing was performed, adjusting for age, to examine the effect of surgical technique on postoperative urinary incontinence, which revealed that NS and Retzius- sparing were significant factors in reducing postoperative urinary incontinence (Table 2, Multivariate 1). A multivariate analysis of each measurement evaluated by MRI, corrected for age, which was significant, showed that longer MUL and shorter PB-AR during abdominal pressure were significant factors in reducing urinary incontinence (Table 2, Multivariate 2).

**Table 2:** Univariate and multivariate logistic regression analysis of urine loss ratio after robot-assisted radical prostatectomy.

Valuables	Univariate		Multivariate 1		Multivariate 2	
	Odds ratio (95%CI)	*P*-value	Odds ratio (95%CI)	*P*-value	Odds ratio (95%CI)	*P*-value
n = 254						
Age, years	1.080(1.027–1.136)	0.003	1,079 (1.021–1.141)	0.007	1.082 (1.020–1.148)	0.008
Body mass index	0.943(0.863–1.029)	0.187				
Prostate specific antigen, ng/ml	0.993(0.963–1.024)	0.653				
D'Amico risk classification						
Low	ref					
Intermediate	0.769 (0.391–1.514)	0.448				
High	1.091 (0.544–2.190)	0.806				
IPSS total score	1.038 (0.996–1.062)	0.077				
ICIQ-UI SF total score	1.069 (0.963–1.188)	0.212				
Nerve-sparing						
Non	ref		ref			
Unilateral	1.733 (0.705–4.259)	0.23	0.301 (0.120–0.757)	0.011		
Bilateral	1.224 (0.585–2.563)	0.592	0.211 (0.067–0.660)	0.008		
PLND						
Non	ref					
Limited	1.505 (0.788–2.873)	0.215				
Exteded	1.449 (0.605–3.473)	0.405				
Retzius-sparing, Yes	0.121 (0.056–0.261)	< 0.001	0.069 (0.027–0.174)	< 0.001		
Removed prostate volume, gr	0.993 (0.976–1.010)	0.427				
Membranous urethral length, mm	0.781 (0.691–0.882)	< 0.001			0.747 (0.645–0.865)	< 0.001
UVJ movement, mm	1.116 (1.037–1.202)	0.003			0.941 (0.854–1.038)	0.225
PB-AR change, mm	0.621 (0.540–0.713)	< 0.001			0.605 (0.515–0.711)	< 0.001

*CI* confidence interval, *ICIQ-UI SF* International Consultation on Incontinence Questionnaire-Urinary Incontinence Short Form, *IPSS* International prostate symptom score, *PB-AR* distance from pubic bone to anterior rectum, *PLND* pelvic lymphnode dissection, *UVJ* urethrovesical junction.

### Distribution of dynamic MRI findings by ULR volume and procedure (C-RARP or RS-RARP)

**Figure 2:**
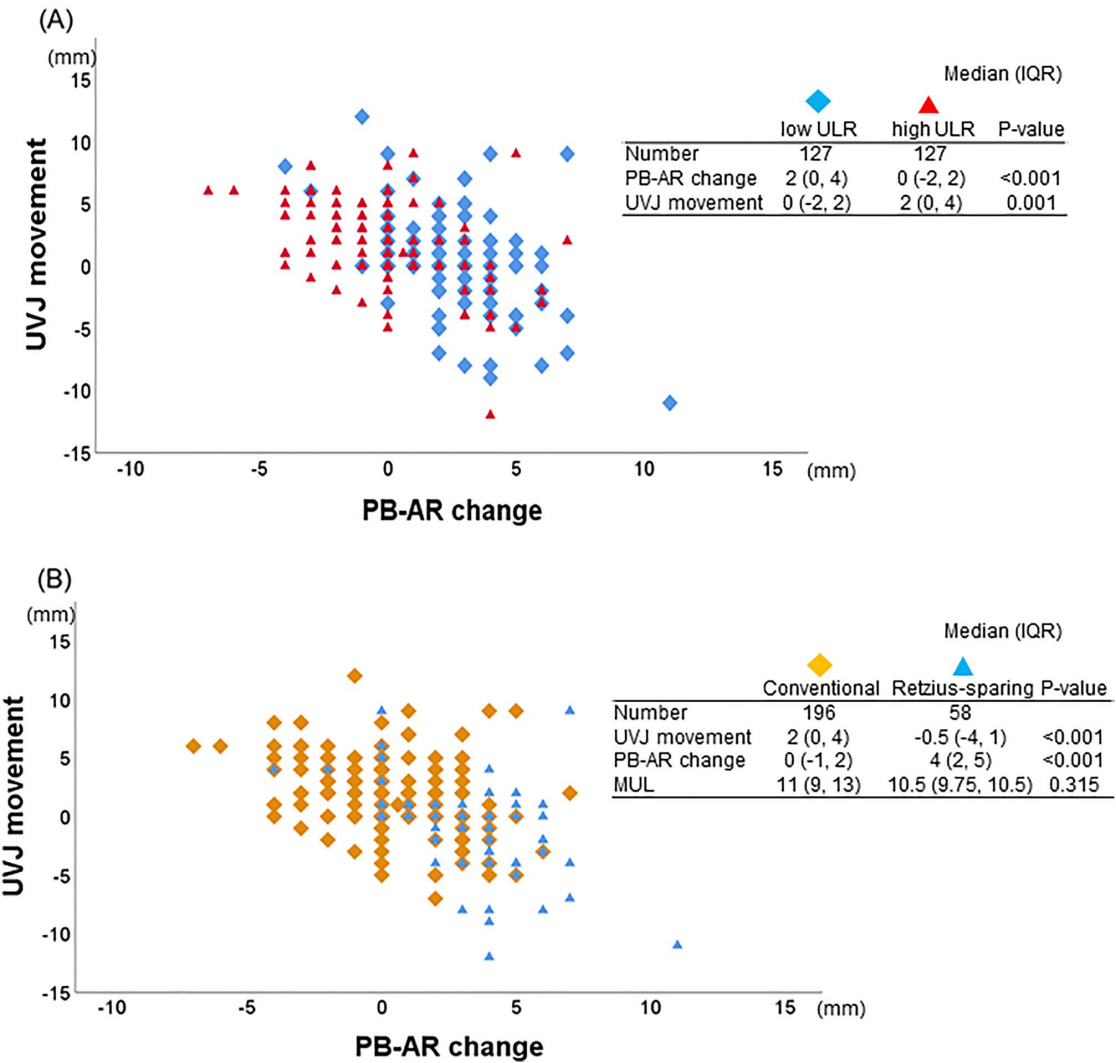
(**A**) Distribution of pubic bone to anterior rectum (PB-AR) change and urethrovesical junction (UVJ) movement for each case by the urine loss ratio (ULR). (**B**) Distribution of PB-AR change and UVJ movement for each case by conventional and Retzius-sparing robot-assisted radical prostatectomy.

Plotting the distribution of PB-AR change and UVJ movement for low and high ULR showed a variation; however, the distribution showed PB-AR shortening due to abdominal pressure and less UVJ movement toward the foot in low ULR cases (Fig. 2A). Plotting the distribution of PB-AR change and UVJ movement for C-RARP and RS-RARP showed a variation; however, the distribution showed PB-AR shortening due to abdominal pressure and less UVJ movement toward the foot in RS-RARP cases, whereas no significant differences were found in MUL between groups (Fig. 2B).

## Discussion

Some recent studies evaluated the changes in pelvic anatomy during abdominal pressure using real-time transperineal ultrasound and dynamic MRI due to the recognition that urinary incontinence after RP is primarily due to SUI^8–10^. The present study investigated the factors that influence the ULR in the early postoperative period using various preoperative and postoperative factors and operative techniques, as well as various postoperative dynamic MRI measurements. Additionally, in the present study, as in previous reports, older age was a risk factor for postoperative urinary incontinence^22–24^ and longer MUL measured by MRI was associated with better urinary continence^3–7^. The results also showed that PB-AR shortening measured by dynamic MRI during abdominal pressure was favorable for urinary continence (Table 2, Multivariate 2). Observation of the external urethral sphincter using a urethroscope during abdominal pressure revealed that the urethral sphincter closes like a shutter in the anterior–posterior direction of the body axis^25^. This mechanism of external urethral sphincter closure during abdominal pressure may be useful in preventing SUI. PB-AR is considered an external urethral sphincter thickness indicator in the anteroposterior direction of the body axis and the shortening of PB-AR during abdominal pressure indicates that it compresses the membranous urethra that runs through its center, which may indicate that its effectiveness in urethral closure. Regarding the influence of surgical technique on postoperative urinary continence, Retzius-sparing and NS were effective in postoperative urinary continence (Table 2, Multivariate 1). The effectiveness of each technique has been previously reported; however, no report has demonstrated that Retzius-sparing and NS are additively effective for postoperative urinary continence (RS-RARP^12–14^, NS^15–17^). The present analysis revealed that NS was not a significant factor in postoperative urinary continence in univariate analysis. The RS-RARP included initial cases among the studied cases, and fewer cases were thought to be treated with combination NS compared to the C-RARP; hence, we adjusted it in the multivariate analysis and found that both had a favorable effect on postoperative urinary continence (Table 2, Multivariate 1). Age, which was significant in the univariate analysis, was also included in the multivariate analysis because it was considered to affect both the surgical technique (Table 2, Multivariate 1) and MRI findings (Table 2, Multivariate 2) when analyzing the effect of postoperative urinary incontinence. The Clinical characteristics of C-RARP and RS-RARP was shown in supplementary Table S1.

A trend toward shorter PB-AR (positive distribution) and smaller UVJ movement (zero to negative distribution) was found during abdominal pressure in the cases with less urinary incontinence when the PB-AR change and UVJ movement were plotted for cases with more and less postoperative urinary incontinence, respectively (Fig. 2A). A significant negative correlation (CC: − 0.488, *p* < 0.001) was found between PB-AR change and UVJ movement despite the wide variation in the distribution of each case. A tendency for PB-AR to shorten (positive distribution) and UVJ movement to decrease (zero to negative distribution) during abdominal pressure was observed in the RS-RARP cases when PB-AR change and UVJ movement were plotted for RS-RARP and C-RARP cases similarly (Fig. 2B). This movement toward shorter PB-AR (positive distribution) and smaller UVJ movement (zero to negative distribution) during abdominal pressure was more frequently observed in RS-RARP cases than in C-RARP cases and may be one of the reasons for less urinary incontinence (Supplement: Video S1). Such movements are likely to occur because the anterior bladder wall is fixed in a higher position in RS-RARP^10^. In transabdominal C-RARP, an incision is made on the peritoneum, and the anterior bladder cavity is opened to approach the prostate. During postoperative reattachment, the anterior bladder wall was fixed more caudally than its preoperative position as the bladder had shifted because of vesicourethral anastomosis. However, even if the peritoneum is sutured following transperitoneal RP or if a retroperitoneal approach is performed without a peritoneal incision, the bladder is pulled so strongly in the caudal direction that when the anterior bladder wall is reattached, the peritoneum is stretched and fixed more caudally than it was preoperatively^10^. No statistical differences were observed in MUL, UVJ movement, or PB-AR change examined in this study depending on NS status (data not shown).

The PB-AR during abdominal pressure is thought to move in the direction of shortening (positive distribution) and UVJ movement in the cephalic direction (zero to negative distribution) when PFMT are effective in urinary continence, and these movements are reportedly effective in urinary continence^8,9,11^. Several RCTs have been conducted on the effect of PFMT on reducing urinary incontinence after RP. A systematic review of RCTs revealed that conditions under which PFMT was effective included preoperative PFMT, combined biofeedback (digital palpation, electromyography), and guidance by a therapist^18^. Variation in individual mastery may have affected the overall PFMT effectiveness, but its mastery is difficult to assess and was not assessed in any RCTs^18^. Reportedly, PFMT guided by transperineal echocardiography was effective in reducing urinary incontinence in patients with prolonged urinary incontinence after RP^26^, and that urinary incontinence was less in patients with a large bladder neck elevation during PFMT as observed by dynamic MRI^9,11^. Teaching PFMT using the image-guided bladder neck elevation and the anterior rectal wall movement toward the pubic bone as a guide may be effective in reducing postoperative urinary incontinence. An RCT that evaluates the efficacy of PFMT with transperineal ultrasound guidance is underway and results are awaited^27^. The present study revealed that the degree of learning varied among cases, which may have contributed to the variation in pelvic floor movement during abdominal pressure on dynamic MRI, although PFMT instruction was preoperatively provided. Additionally, urinary incontinence was also less in cases in which movement was observed in the direction of shortening PB-AR (positive distribution) and small UVJ movement (zero to negative distribution) during abdominal pressure, which suggests that PFMT may have been effectively acquired in these cases.

Many studies reported on factors that affect urinary incontinence after RP. The present study identified older age^22–24^, and shorter MUL^3–7^ as worsening factors for postoperative urinary incontinence, as has been previously reported. Regarding operative technique, NS^15–17^ and Retzius-sparing^12–14^ were identified as favorable factors for postoperative urinary continence, and their combined use was shown to have an additive urinary continence effect. Reportedly, leaving the urethra as long as possible has a favorable effect on postoperative urinary continence^28^, and we try to preserve as much urethral length as possible in our clinic. Maintaining urethral closure pressure at rest and preserving the mechanisms that effectively work when intravesical pressure increases during abdominal pressure is necessary to maintain favorable urinary continence, and not losing these functions will be important to maintain urinary continence after RP. Preserving a longer urethra^28^ and NS^17^ are useful to avoid losing urethral closure pressure at rest after RP, and Retzius-sparing^10^ and PFMT instruction with biofeedback, such as imaging^9,11,26^, may contribute to urinary continence mechanism preservation during abdominal pressure. The patient’s potential may also have an effect on the original urinary sphincter function, and age is probably representative of this^22–24^. Every single factor alone probably has only a small effect on urinary continence after RP, and the final urinary continence outcome will need to consider them in combination in several case-to-case variations.

Moreover, this study has limitations. First, the dynamic MRI was performed in the supine position, and the patient was not evaluated in the standing position. Additionally, the patient was instructed to apply abdominal pressure during this dynamic MRI; however, it did not faithfully reproduce the sudden sneezing and applied abdominal pressure that would normally be expected to predispose the patient to SUI. The PB-AR changes that are observed on dynamic MRI may affect the closure pressure of the membranous urethra, but the actual extent of the change in closure pressure has not been measured. The PFMT in this study used pamphlets and only verbal explanations by staff, and no biofeedback was provided, suggesting that individual differences are present in the mastery degree.

## Conclusions

Pelvic anatomy on urinary continence at rest and during abdominal pressure was investigated using dynamic MRI. Long MUL and an effective urethral sphincter closure mechanism during abdominal pressure were considered as effective for favorable urinary continence after RP. NS and Retzius-sparing were shown as effective for urinary incontinence, and their combination was clearly shown to have an additive effect in preventing urinary incontinence for the first time. Urinary continence was thought to be better in cases in which the PFMT has mastered the technique of adequately compressing the urethral sphincter during abdominal pressure.

## Supplementary Information

Supplementary Table S1.

Supplementary Information.

Supplementary Video S1.

## Data Availability

The datasets used and/or analyzed during the current study available from the corresponding author on reasonable request.

## Abbreviations

CC: Correlation coefficient
C-RARP: Conventional robot-assisted radical prostatectomy
MUL: Membranous urethral length
MV: Micturition volume
NS: Nerve-sparing
PB-AR: Distance from the pubic bone to the anterior rectum
PC: Prostate cancer
PFMT: Pelvic floor muscle training
QOL: Quality of life
RARP: Robot-assisted radical prostatectomy
RCT: Randomized controlled trial
RP: Radical prostatectomy
RS-RARP: Retzius-sparing robot-assisted radical prostatectomy
SUI: Stress urinary incontinence
UL: Urine loss
ULR: Urine loss ratio
UVJ: Urethrovesical junction
