# Supplementary material for: Contribution of Retzius-sparing robot-assisted radical prostatectomy to the mechanism of urinary continence as demonstrated by dynamic MRI
Source: Sci Rep. 2023 Feb 18;13:2902. doi: 10.1038/s41598-023-30132-x (PMC9938893; doi:10.1038/s41598-023-30132-x)
Supplement: Supplementary file 1 — Supplementary Table S1. [file 41598_2023_30132_MOESM1_ESM.pdf]

Table S1. Clinical characteristics of conventional and Retzius-sparing robot-assisted radical prostatectomy

| | | Median (IQR) or n (%) | |
|---|---|---|---|
| | C-RARP | RS-RARP | P-value |
| Number of patients | 196 | 58 | |
| Age, years | 66（63-70） | 67 (62-72) | 0.913 |
| Body mass index | 23.5（21.7-24.7） | 21.7 (20.9-23.7) | 0.779 |
| Prostate specific antigen, ng/ml | 7.1（5.5-9.5） | 9.3 (5.0-21.5) | 0.400 |
| Biopsy Gleason Grade Group | | | 0.807 |
| 1 | 44 (22%) | 10 (17%) | |
| 2 | 55 (28%) | 17 (30%) | |
| 3 | 41 (21%) | 16 (28%) | |
| 4 | 48 (25%) | 13 (22%) | |
| 5 | 8 (4%) | 2 (3%) | |
| Clinical stage | | | 0.714 |
| ≦T2 | 191 (97%) | 56 (97%) | |
| T3≦ | 5 (3%) | 2 (3%) | |
| D'Amico risk classification | | | 0.651 |
| low | 39 (20%) | 9 (16%) | |
| intermediate | 87 (44%) | 25 (43%) | |
| high | 70 (36%) | 24 (41%) | |
| NADT | | | 0.447 |
| No | 180 (92%) | 55 (23%) | |
| Yes | 16 (8%) | 3 (5%) | |
| IPSS total score | 10 (6-17) | 11 (9-16) | 0.159 |
| ICIQ-UI SF total score | 0 (0-3) | 2 (0-7) | 0.243 |
| Nerve-sparing | | | <0.001 |
| non | 24 (12%) | 21 (36%) | |
| unilateral | 141 (72%) | 34 (59%) | |
| bilateral | 31 (13%) | 3 (5%) | |
| *Surgical time, min. | 257 (225-278) | 239 (208-283) | <0.001 |
| *Console time, min. | 203 (167-223) | 172 (144-242) | <0.001 |
| PLND | | | <0.001 |
| non | 129 (66%) | 55 (95%) | |
| limited | 47 (24%) | 0 (0%) | |
| exteded | 20 (10%) | 3 (5%) | |
| Bleeding, mL | 60 (30-150) | 150 (38-275) | <0.001 |
| Removed prostate volume, gr. | 37.0（32.0-50.0） | 32.0 (30.3-34.0) | 0.415 |
| Catheter indwelling duration, days | 7 (7-7) | 7 (7-7) | 0.169 |
| Urine loss ratio, % | 6.3 (0.9-18.4) | 0.4 (0.0-4.5) | 0.030 |

C-RARP, conventional robot-assisted radical prostatectomy; IQR, interquartile range; ICIQ-UI SF, International Consultation on Incontinence Questionnaire-Urinary Incontinence Short Form; IPSS, International prostate symptom score; NADT, neoadjuvant androgen deprivation therapy; PLND, pelvic lymphnode dissection; RS-RARP, Retzius-sparing robot-assisted radical prostatectomy.
*Surgical time and Console time include PLND time.
