# Supplementary material for: Contribution of Retzius-sparing robot-assisted radical prostatectomy to the mechanism of urinary continence as demonstrated by dynamic MRI
Source: Sci Rep. 2023 Feb 18;13:2902. doi: 10.1038/s41598-023-30132-x (PMC9938893; doi:10.1038/s41598-023-30132-x)
Supplement: Supplementary file 2 — Supplementary Information. [file 41598_2023_30132_MOESM2_ESM.docx]

**Video S1. Dynamic MRI findings.**

Dynamic MRI showing pelvic anatomical changes during the abdominal pressure application after conventional and Retzius-sparing robot-assisted radical prostatectomy.

(Narration is created by ondoku3.com.)
